# Reduction of dynamical biochemical reactions networks in computational biology

**DOI:** 10.3389/fgene.2012.00131

**Published:** 2012-07-19

**Authors:** O. Radulescu, A. N. Gorban, A. Zinovyev, V. Noel

**Affiliations:** ^1^DIMNP UMR CNRS, University of Montpellier 2Montpellier, France; ^2^Department of Mathematics, University of LeicesterLE, UK; ^3^Institut Curie, INSERM/Curie/Mines ParisTechParis, France; ^4^IRMAR UMR, University of Rennes 1Rennes, France

**Keywords:** computational biology, dynamical networks, model reduction, quasi-equilibrium approximation, quasi-steady state approximation

## Abstract

Biochemical networks are used in computational biology, to model mechanistic details of systems involved in cell signaling, metabolism, and regulation of gene expression. Parametric and structural uncertainty, as well as combinatorial explosion are strong obstacles against analyzing the dynamics of large models of this type. Multiscaleness, an important property of these networks, can be used to get past some of these obstacles. Networks with many well separated time scales, can be reduced to simpler models, in a way that depends only on the orders of magnitude and not on the exact values of the kinetic parameters. The main idea used for such robust simplifications of networks is the concept of dominance among model elements, allowing hierarchical organization of these elements according to their effects on the network dynamics. This concept finds a natural formulation in tropical geometry. We revisit, in the light of these new ideas, the main approaches to model reduction of reaction networks, such as quasi-steady state (QSS) and quasi-equilibrium approximations (QE), and provide practical recipes for model reduction of linear and non-linear networks. We also discuss the application of model reduction to the problem of parameter identification, via backward pruning machine learning techniques.

## 1. Introduction

During the last decades, biologists have identified a wealth of molecular components and regulatory mechanisms underlying the control of cell functions. Cells integrate external signals through sophisticated signal transduction pathways, ultimately affecting the regulation of gene expression, including that of the signaling components. Metabolic functions are sustained and controlled by complex machineries involving genes, enzymes, and metabolites. The genetic regulations result from the coordinate effect of many, mutually interacting genes. These regulations involve many molecular actors, including proteins and regulatory RNAs, which form large, intricate networks.

Current dynamical models of cellular molecular processes are small size networks. These small scale models, that are subjective simplifications of reality, can not take into account the specificities of regulatory mechanisms. New methods are needed, allowing to reconcile small scale dynamical models and large scale, but static, network architectures. The main obstacle to increasing the size of dynamical networks is the incomplete information, on the parameters and on the mechanistic details of the interactions. *In vivo* values of the parameters depend on crowding and heterogeneity of the intracellular medium, and can be orders of magnitude different from what is measured *in vitro*. Furthermore, learning models from data suffer for non-identifiability and over-fitting problems. Thus, model reduction is an avoidable step in the study of large networks, allowing to extract the essential features of the model, that can then be identified from data. Model reduction in computational biology should have several features.

First of all, model reduction should cope with parametric incompleteness and/or uncertainty.

A certain class of reduction methods are parameter independent and automatically comply with this specificity. In biochemical networks, the number of possible chemical species grows combinatorially due to numerous possibilities of interactions between molecules with multiple interaction sites. The exact lumping methods (Borisov et al., 2005; Conzelmann et al., 2006) reduce the number of microstates and avoid combinatorial explosion in the description and analysis of large models of receptor and scaffold signaling. A similar technique (Feret et al., 2009) is used to rationally organize supramolecular complexes in rule-based modeling (Danos et al., 2007a) of biochemical networks. Other, parameter independent, coarse-graining techniques are graphical methods formalizing node deletion and merging operations in biochemical networks (Gay et al., 2010), pooling of metabolites in large scale metabolic networks (Papin et al., 2004; Jamshidi and Palsson, 2008), or extensive searches in the set of all possible lumps (Dokoumetzidis and Aarons, 2009). Finally, qualitative reduction methods were used to simplify large logical regulatory graphs, adequately suppressing nodes and defining sub-approximating dynamics (Naldi et al., 2009, 2011).

Secondly, biochemical processes governing network dynamics span over many timescales. For example, changing gene expression programs can take hours and even days while protein complex formation goes on the second scale and post-translational protein modifications take minutes to happen. Protein life half-times can vary from minutes to days. Model reduction can strongly benefit from the network multiscaleness. Asymptotic dynamics of networks with slow and fast processes, can be strongly simplified using various ideas such as inertial and invariant manifolds (IM) and averaging approximations.

The iterative methods of IM aim to find a slow low dimensional IM, containing the asymptotic dynamics (Gorban and Karlin, 1994, 2003; Roussel and Fraser, 1991). The Computational Singular Perturbation (CSP) (Lam and Goussis, 1994; Chiavazzo et al., 2007) aims to find even more, the slow IM and, in addition, the geometry of its fast foliation. IM can be calculated by various other methods (Gorban and Karlin, 2005; Gorban et al., 2004; Roussel and Fraser, 1991; Kazantzis and Good, 2002; Krauskopf et al., 2005).

Very popular are the methods for computation of “first approximations” to the slow IM. The classical quasi steady-state approximation (QSS) was proposed by Bodenstein (1913) and was elaborated into an important tool for analysis of chemical reaction mechanism and kinetics (Semenoff, 1939; Christiansen, 1953; Helfferich, 1989). The classical QSS is based on the relative smallness of concentrations of some of active reagents (radicals, concentration of enzyme and substrate-enzyme complexes, or amount of active centers on the catalyst surface) (Aris, 1965; Segel and Slemrod, 1989; Yablonskii et al., 1991). The quasi-equilibrium approximation (QE) has two basic formulations: the thermodynamic approach, based on conditional entropy maximum (or free energy conditional minimum), or the kinetic formulation, based on equilibration of fast reversible reactions. The very first use of the entropy maximum dates back to Gibbs (Gibbs, 2010). Corrections to QE approximation with applications to physical and chemical kinetics were developed by (Gorban et al., 2001; Gorban and Karlin, 2005). An important, still unsolved, problem of these two approximations is the detection of QSS species and QE reactions without application of all machinery of the IM or CSP methods. Indeed, not all reactions with large constants are at QE, and there are no simple rules to find QSS species if there is no such hints as a small amount of a conserved quantity (like the total concentration of enzyme). The method of Intrinsic Low Dimensional Manifolds (ILDM) (Maas and Pope, 1992; Bykov et al., 2006) provides an approximation of a low dimensional IM and works as a first step of CSP (Kaper and Kaper, 2002).

Another method allowing to simplify multiscale dynamics is averaging. This idea can be tracked back to Poincaré's perturbative treatment of the many body problem in celestial mechanics (Poincarè, 1899), further developed in classical mechanics by other authors (Arnold, 1978; Lochack and Meunier, 1988), and also known as adiabatic or Born-Oppenheimer approximation in quantum mechanics (Messiah, 1962). Rather generally, averaging can be applied when some fine scale variables of the system are rapidly oscillating. Then, the dynamics of slow, coarse scale variables, can be obtained by time averaging the system over a timescale much larger than the period of the fast oscillations. The way to perform averaging, depends on the structure of the system, namely on the definition of the coarse grained and fine variables (Bogoliubov and Mitropolski, 1961; Artstein and Vigodner, 1996; Givon et al., 2004; Acharya and Sawant, 2006; Sawant and Acharya, 2006; Acharya, 2010; Slemrod, 2011).

Some of these ideas have been implemented in computational biology tools. Systems biology markup language SBML (Hucka et al., 2003) can allocate a “fast” attribute to reaction elements. Fast reaction specification can be taken into account by computational biology softwares such as VirtualCell (Slepchenko et al., 2003) that implements a QE approximation algorithm (Slepchenko et al., 2000). Similarly, the simulation tool COPASI (Hoops et al., 2006) implements the ILDM method (Surovtsova et al., 2009).

Finally, multiscaleness does not uniquely apply to timescales, but equivalently to abundances of various species in these networks. mRNA copy numbers can change from some units to tens of thousands, and the dynamic concentration range of biological proteins can reach up to five orders of magnitude. Furthermore, the DNA molecule has only one or a few copies. Low copy numbers lead, directly or indirectly (a species can be stochastic even if present in large copy numbers), to stochastic gene expression. In computational biology, model reduction should thus cope not only with deterministic, but also with stochastic and hybrid models. The need to reduce large scale stochastic models is acute. Indeed, stochastic simulation algorithm (SSA, Gillespie, 1976, 1977) can be very expensive in computer time when applied to large unreduced models, precluding model analysis and identification. For this reason, extensive effort has been dedicated to adapting the main ideas used for model reduction of deterministic models, namely exact lumping, IM, QSS, QE, and averaging, to the case of stochastic models.

Reduction of stochastic rule-based models, based on a weakened version of the exact lumpability criterion, has been proposed by Feret et al. (2012) to define abstract species or stochastic-fragments that can be further used in simplified calculations. More generally, rule-based models alow to overcome combinatorial complexity in stochastic simulations (Danos et al., 2007b). The performance of rule-based stochastic simulators such as NFsim (Sneddon et al., 2011) scales independently of the reaction network size. Approximate reduction of the number of states of the Markov chains describing stochastic networks were proposed in Munsky and Khammash (2006).

Multiscaleness of stochastic networks is two-fold, it affects both species and reaction rates. This has been exploited in hybrid stochastic simulation schemes that are, for the most of them, based on a partition of the biochemical reactions in fast and slow reactions (Haseltine and Rawlings, 2002; Burrage et al., 2004; Alfonsi et al., 2005; Haseltine and Rawlings, 2005; Alfonsi et al., 2004; Salis and Kaznessis, 2005; Kaznessis, 2006; Harris and Clancy, 2006; Surovtsova et al., 2006; Salis et al., 2006; Griffith et al., 2006; Ball et al., 2006; Li et al., 2008; Gómez-Uribe et al., 2008; Pahle, 2009). Conversely, mixed partitions, using both reactions and species can exploit both types of multiscaleness and more appropriately unravel a rich variety of stochastic functioning regimes such as piece-wise deterministic, switched diffusions, diffusions with jumps, as well as averaged processes (Radulescu et al., 2007; Crudu et al., 2009, 2012) only partially covered by some situations discussed in Mastny et al. (2007).

Machine learning approaches to parameter identification (Golightly and Wilkinson, 2011) could profit from Fokker–Planck approximations, also known as diffusion approximations or Langevin approach, of the master equation describing dynamics of stochastic networks. Traditional approaches such as central limit theorem (Gillespie, 2000; Mélykúti et al., 2010), the Ω and the Kramers–Moyal expansions (Radulescu et al., 2007; Crudu et al., 2009) where used to derive diffusion approximations. Alternatively, (Erban et al., 2006) propose diffusion approximations for slow/fast stochastic networks, in which the drift and diffusion parameters were obtained numerically. More recently, these parameters were derived directly from the master equation of stochastic networks with species in small and large copy numbers (Radulescu et al., 2012). Furthermore, by the ergodic theorem, time averaging of multiscale stochastic models boils down to a QE assumption for the fast variables. This idea has been used in Crudu et al. (2009) to reduce stochastic networks. A few computational biology tools implement stochastic approximations (Salis et al., 2006).

With the exception of the parameter independent methods, all the model reduction methods described above need a full parametrization of the model. This is a stringent requirement, and can not be easily bypassed. Indeed, the reduction has a local validity. The elements defining a reduced model such as IM, QSS species, QE species, depend on the model parameters and also on the position in phase space and along trajectories. What one can expect is that model reduction is robust, i.e., a given reduced model provides an accurate approximation of the dynamics of the initial model for a wide range of parameters and variables values. One can show that this property is satisfied by biochemical networks with separated constants, because in this case the simplified networks depend on the order relations among model parameters and not on the precise values of these parameters (Gorban and Radulescu, 2008; Radulescu et al., 2008; Noel et al., 2011).

The purpose of this review is not the exhaustive description of all the reduction methods that we have delineated. We will revisit the fundamental concepts of model reduction in the light of a new framework, that should, in the long-term, lead to a new generation of reduction tools satisfying all the specific requirements of computational biology. Due to space limitations, we restrict ourselves to deterministic models.

## 2. Deterministic dynamical networks

To construct a dynamic reaction network we need the list of components, $A=\left\{A_{1},\ldots A_{n}\right\}$ and the list of reactions (the reaction mechanism):

(1) $$\sum_{1}\alpha_{ji}A_{i}⇌\sum_{k}\beta_{jk}A_{k},$$

where *j* ∈ [1, *r*] is the reaction number.

Dynamics of non-linear networks in homogeneous isochoric systems (fixed volume) is described by a system of differential equations:

(2) $$\frac{dc}{dt}=P\left(c\right)=\sum_{j=1}^{r}\nu_{j}\left(R_{j}^{+}\left(c\right)-R_{j}^{-}\left(c\right)\right)$$

*c* ∈ ℝ^*n*^ is the concentration vector, ν_*j*_ = β_*j*_ − α_*j*_ is the global stoichiometric vector. The reaction rates *R*^+/−^_*j*_(*c*) are non-linear functions of the concentrations. For instance, the mass action law reads *R*^+^_*j*_(*c*) = *k*^+^_*j*_ ∏_*i*_ *c*^α_*ji*_^_i_, *R*^−^_*j*_(*c*) = *k*^−^_*j*_ ∏_*i*_ *c*^β_*ji*_^_i_, in which case *P*_*i*_(*c*) is a multivariate polynomial on the concentrations *c*_*j*_.

## 3. Multi-scale reduction of monomolecular reaction networks

Monomolecular reaction networks are the simplest reaction networks. The structure of these networks is completely defined by a digraph, in which vertices correspond to chemical species *A*_*i*_, edges correspond to reactions *A*_*i*_ → *A*_*j*_ with kinetic constants *k*_*ji*_ > 0.

The kinetic equation is

(3) $$\frac{dc_{i}}{dt}=\sum_{j}k_{ij}c_{j}-\left(\sum_{j}k_{ij}\right)c_{i,}\;\;\;\;i\;\in \left[1,n\right],$$

or in matrix form: $\dot{c}=Kc$.

The solutions of (3) can be expressed in terms of left and right eigenvectors of the kinetic matrix ***K***:

(4) $$c\left(t\right)=\left(l^{0},c\left(0\right)\right)+\sum_{k=1}^{n-1}r^{k}<l^{k},c\left(0\right)>\exp\left(-\lambda_{k}t\right)$$

where ***Kr***^*k*^ = λ_*k*_ ***r***^*k*^, and ***l***^*k*^ ***K*** = λ_*k*_ ***l***^*k*^.

Each eigenvalue λ_*k*_ is the inverse of a timescale of the network. A reduced network having solutions of the type (4), with eigenvectors ***r***^*k*^, ***l***^*k*^, and eigenvalues λ_*k*_ approximating the eigenvectors and the eigenvalues of the original network is called a *multiscale approximation*.

We say that the network constants are totally separated if for all (*i, j*) ≠ (*i*′,*j*′) one of the relations *k*_*ji*_ << *k*_*j*′*i*′_, or *k*_*ji*_ >> *k*_*j*′*i*′_ is satisfied.

It was shown in Gorban and Radulescu (2008); Radulescu et al. (2008); Gorban et al. (2010) that the multiscale approximations of arbitrary monomolecular reaction networks with totally separated constants are acyclic (have no cycles), and deterministic (have no nodes from which leave more than one edge) digraphs.

In order to reduce a network with total separation, one needs only qualitative information on the constants. More precisely, each edge of the reaction digraph can be labeled by a positive integer representing the rank of the reaction parameter in the ordered series of parameter values, the largest parameter (the quickest reaction) having the lowest label. These integer labels also indicate the timescales of the processes modeled by the network reactions.

**Figure 1:**
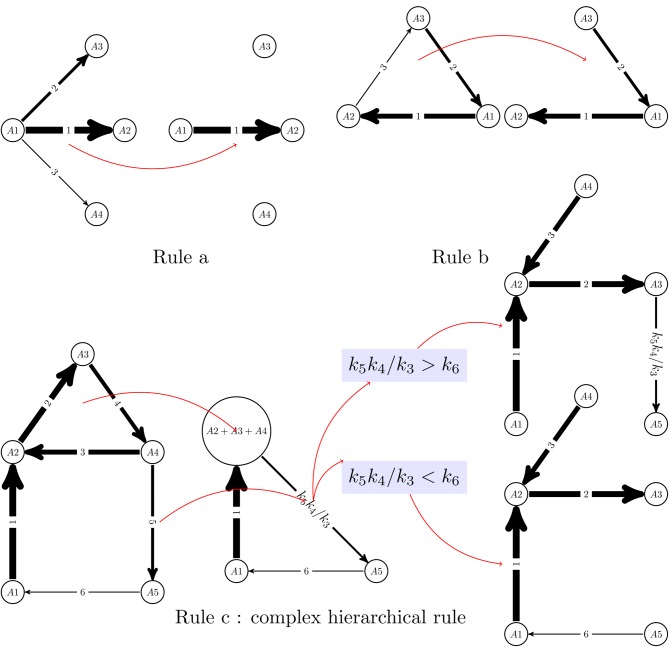
**Reduction algorithm for linear networks.** A monomolecular network with total separation can be represented as a digraph with integer labels (the quickest reaction has label 1). Two simple rules allow to eliminate competition between reactions (rule a) and transform cycles into chains (rule b). Rule b can not be applied to cycles with outgoing slow reactions, in which case more complex, hierarchical rules should be applied (rule c). In the rule c, first the cycle *A*_2_ → *A*_3_ → *A*_4_ → *A*_2_ is “glued” to a new node (pool *A*_2_+ *A*_3_+ *A*_4_) and the constant of the slow outgoing reaction renormalized to a monomial *k*_5_*k*_4_/*k*_3_. Rule b is applied to the resulting network, which is a cycle with no outgoing reactions. The comparison of the constants *k*_5_*k*_4_/*k*_3_ and *k*_6_ dictates where this cycle is cut. Finally, the glued cycle is restored, with its slowest step removed.

The reduced network is not always a subgraph of the initial graph. It is obtained from this integer labeled digraph by graph re-writing operations, that can be generically described as pruning and pooling. Two types of pruning operations are of primary importance (see also Figure 1):

**Rule a)** If one has one node from which leave more than one edge, then all the edges are pruned with the exception of the fastest one (lowest integer label). This operation corresponds to keeping the dominant term among the terms *c*_*i*_*k*_*ij*_ consuming a species *A*_*i*_, and reduces the node outdegree to one. The same principle can not be applied to reduce the indegree, because which production term is dominant among *k*_*ij*_ *c*_*j*_, *j* ∈ [1,*n*], depends not only on *k*_*ij*_ but also on the concentrations *c*_*j*_.

**Rule b)** Cycles with separated constants can be transformed into chains, by elimination of the slowest step. This can be justified intuitively by topology, because any two nodes of a cycle are connected by two paths, one containing the slowest step and the other one not containing the slowest step. The latter shortcuts the former.

However, a combination of rules a) and b) is not allowed to prune slow reactions leaving a cycle and further transform the cycle into a chain by eliminating the limiting step. Indeed, the total mass of such cycles is slowly decaying because of outgoing reactions. Pruning the slow reactions that leave a cycle would keep the total cycle mass constant and produce the wrong long time approximation. In this case, pooling operations are needed:

**Rule c)** Glue each cycle in the pruned system into a new vertex and transform the network of *all initial reactions* into a new one. The concentration of this new component is the sum of the concentration of the glued vertices. Reactions to the cycles transform into reactions to the correspondent new vertices (with the same constants). To transform the reactions from the cycles, we have to calculate the normalized quasi-stationary distributions inside each cycle (with unit sum of the concentrations in each cycle). Let for the vertex *A*_*i*_ from a cycle this concentration be *c*^o^_*i*_. Then the reaction *A*_*i*_ → *A*_*j*_ with the constant *k*_*ji*_ transforms into the reaction from the new (“cycle”) vertex with the constant *k*_*ji*_ *c*^o^_*i*_. The destination vertex of this reaction is *A*_*j*_ if it does not belong to a cycle of the pruned system, it is the correspondent glued cycle if it includes *A*_*j*_ and does not include *A*_*i*_ and the reaction vanishes if both *A*_*i*_ and *A*_*j*_ belong to the same cycle of the pruned system.

After pooling we have to prune (Rule a) and so on, until we get an acyclic pruned system. Then the way back follows: we have to restore cycles and cut them (Rule b).

**Figure 2:**
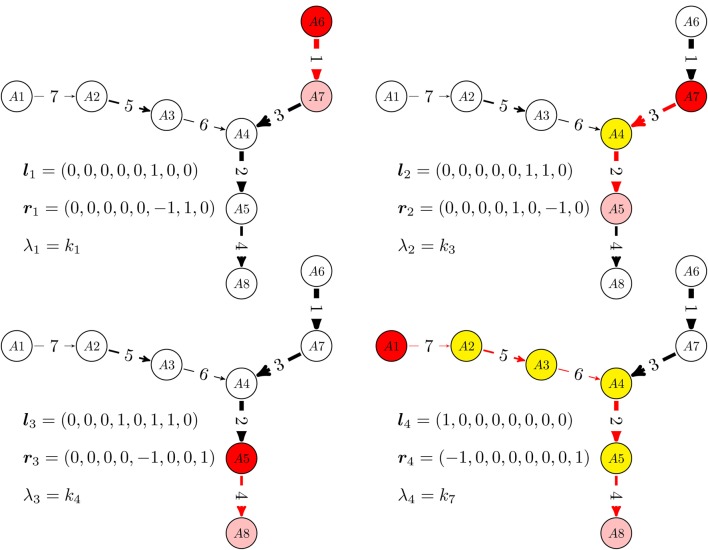
**Relaxation modes of linear multiscale networks.** For a given timescale, monomolecular networks with total separation behave as a single step: the concentrations of some species (white) are practically constant, some species (yellow) are rapid, low concentration, intermediates, one species (red) is gradually consumed and another (pink) is gradually produced. We have represented the sequence of one step approximations of a reduced, acyclic, deterministic digraph, from the quickest time-scale *t*_1_ = λ^−1^_1_ to the slowest one *t*_4_ = λ^−1^_4_. These one step approximations are activated when mass is introduced at *t* = 0 via the “boundary nodes” *A*_1_ and *A*_6_.

In more detail, the graph re-writing operations, are described in the Appendix and illustrated in Figure 1. The dynamics of reduced acyclic deterministic digraphs follows from their topology and from the timescale labels. First of all, let us notice that the network has as many timescales as remaining edges in the reduced digraph. The computation of eigenvectors of acyclic deterministic digraphs is straightforward (Gorban and Radulescu, 2008; Radulescu et al., 2008; Gorban et al., 2010). For networks with total separation, these eigenvectors satisfy, in the first approximation, a 0−1 type property, the coordinates of ***l***^*k*^, ***r***^*k*^ belong to the sets {0, 1}, and {0, 1, −1}, respectively. The 0−1 property of eigenvectors has a non-trivial consequence. On the timescale *t*_*k*_ = (λ_*k*_)^−1^, the reduced digraph behaves as an effective reaction (single step approximation). The effective reaction receives (from reactions acting on smaller timescales) the mass coming from the species with coordinate 1 in ***l***^*k*^ (pool) and transfers it (during a time *t*_*k*_) to the species with coordinate 1 in ***r***^*k*^. The successive single step approximations of an acyclic deterministic digraph are illustrated in Figure 2.

Monomolecular networks with separation represent instructive examples where reduction and qualitative dynamics result from the network topology and from the orders of magnitude of the kinetic constants. This type of models can be used in computational biology to reduce linear subnetworks or even binary reactions for which one reactant is present in much larger quantities than the other (pseudo-monomolecular approximation).

As argued by a few authors, total separation could be a generic property of biochemical networks (Furusawa and Kaneko, 2003). This property can be checked empirically by investigating the distribution of network timescales in logarithmic scale. Whenever one finds distributions with large support in logarithmic scale a log-uniform distribution is equivalent to the Zipf law, i.e., a power law distribution with exponent −1, well known in critical systems (Furusawa and Kaneko, 2003) total separation is valid and the above reduction method applies.

## 4. Separation, dominance, and tropical geometry

The previously presented algorithm is based on the idea of dominance, which occurs at many levels. For instance, when several reactions compete for the same pool, all can be pruned, excepting the dominant one [Rule a]. This simple idea is widely spread, and corresponds to max-plus algebra: the sum of positive, well separated terms, can be replaced by the maximum term. Max-plus algebra, that found many applications to dynamical systems (Cohen et al., 1999; van den Boom and De Schutter, 2006; Aubin, 2010), belong to the new mathematical field of tropical geometry (Pachter and Sturmfels, 2004). Tropical geometry offers convenient solutions to finding approximate roots of simultaneous polynomial equations, as well as to simplifying and hybridizing systems of polynomial or rational ordinary differential equations with separated monomials. Tropical geometry concepts can be used to rationalize many model reduction operations and find new ones.

The logarithmic transformation *u*_*i*_ = log *x*_*i*_, 1 ≤ *i* ≤ *n*, well known for drawing graphs on logarithmic paper, plays a central role in tropical geometry (Viro, 2008).

Let us consider multivariate monomials *M*(*x*) = *a*_α_*x*^α^, where *x*^α^= *x*^α_1_^_1_ *x*^α_2_^_2_ … *x*^α_*n*_^_*n*_. Monomials with positive coefficients *a*_α_>0, become linear functions, log *M* = log *a*_α_ + <α, log(*x*)>, by this transformation.

There is a straightforward way to use the logarithmic transformation from tropical geometry in order to obtain approximations of dynamical networks of the type (2). Let us suppose that reaction rates are polynomial functions of the concentrations (this is satisfied by mass action law and obviously, also by monomolecular networks), such that ∑^*r*^_j=1_ ν_*j*_(*R*^+^_*j*_(*c*) − *R*^−^_*j*_(*c*)) = ∑_α ∈ *A*_ *a*_α_ *c*^α^, *A* ⊂ ℕ^*n*^.

We call tropicalization of the smooth ODE system (2) the following piecewise-smooth system:

(5) $$\frac{dc_{i}}{dt}=s_{i}\exp\left[\max_{\alpha \in A_{i}}\left\{\log\left(\left|a_{i,\alpha }\right|\right)+<\log\left(c\right),\alpha >\right\}\right],$$

where log(*c*) = (log *c*_1_, …, log *c*_*n*_), *s*_*i*_ = *sign*(*a*_*i*,α_max__) and *a*_*i*,α_max__, α_max_∈ *A*_*i*_ denotes the coefficient of a monomial for which the maximum occurring in (5) is attained.

The tropicalization associates to a polynomial ∑_α ∈ *A*_ *a*_α_ *c*^α^, the max-plus polynomial

$$P^{\tau }\left(c\right)=\exp\left[\max_{\alpha \in A}\left\{\log\left(\left|a_{\alpha }\right|\right)+<\log\left(c\right),\alpha >\right\}\right],$$

**Figure 3:**
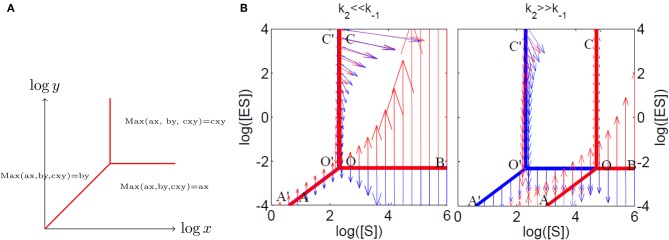
**Tropical geometry and Michaelis–Menten mechanism. (A)** The tropical manifold of the polynomial *ax* + *by* + *cxy* on “logarithmic paper” is a three lines tripod. **(B)** The tropical manifolds for the species ES (in red) and S (in blue) for the Michaelis–Menten mechanism. The tropicalized flow is also represented on both sides of the tropical manifolds (with arrows, red on one side, blue on the other side). Sliding modes correspond to blue and red arrows pointing in opposite directions.

In other words, a polynomial is replaced by a piecewise smooth function, equal to the largest, in absolute value, of its monomials. Thus, (5) is a piecewise smooth model (Naldi et al., 2011; Noel et al., 2011, 2012) because the dominating monomials in the max-plus polynomials can change from one domain to another of the concentration space. The singular set where at least two of the monomials are equal, and where the max-plus polynomial *P*^τ^(*c*) is not smooth is called tropical manifold (Mikhalkin, 2007). On logarithmic paper, the tropical manifolds of various species define polyhedral domains inside which the dynamics is defined by monomial differential equations (Figure 3). Tropicalized systems remind of, but are not equivalent to, Savageau's S-systems (Savageau and Voit, 1987) that have been used for modeling metabolic networks. S-systems are smooth systems such that the production and consumption terms of each species are multivariate monomials. Tropicalized systems are S-systems locally, within the polyhedral domains defined by the tropical manifolds, and also along some parts of the tropical manifold (that carry sliding modes, see next section).

The tropicalization unravels an important property of multiscale systems, that is to have different behavior on different timescales. We have seen that, on every timescale, monomolecular networks with total separation behave like a single reaction step. This is akin to considering only the dominant processes in the network and implies that the tropicalization is a good approximation for monomolecular networks with total separation.

The tropical geometry framework is particularly interesting for non-linear networks. In this case, it is less straightforward to define separation rigorously. Very roughly, one can say that a system (2) with polynomial rates is separated, if the monomials composing the rates are separated almost all the time on a trajectory, or, equivalently, almost everywhere in phase space (except on the tropical manifolds). Separation of non-linear models results either from separated kinetic constants, or from separated species concentrations, or both. In the next section, we discuss some examples when the tropicalization provides useful approximations of smooth non-linear networks.

## 5. Quasi-steady state and quasi-equilibrium, revisited

Two simple methods are principally useful for model reduction of non-linear models with multiple timescales: the quasi-equilibrium (QE) and the quasi-steady state (QSS) approximations. As discussed in Gorban et al. (2010); Gorban and Shahzad (2011), these two approximations are physically and dynamically distinct. In order to understand these differences let us refer to the simple example of the Michaelis–Menten mechanism,

(6) $$S+E\underset{k_{-1}}{\overset{k_{1}}{⇌}}ES\overset{k_{2}}{\to }P+E$$

The QSS approximation, proposed for this system by Briggs and Haldane, considers that the total concentration of enzyme, *E*_tot_=[*E*]+[*ES*], is much lower than the total concentration of substrate, therefore, the complex *ES* is a *low concentration, fast species.* The complex concentration is slaved by the concentration of *S*, meaning that the value of [*ES*] almost instantly relaxes to a value depending on [*S*]. The simplified mechanism correspond to pooling the two reactions of the mechanism into a unique irreversible reaction $S\overset{R([S],E_{tot})}{\to }P,$ which means that $\frac{d[P]}{dt}=-\frac{d[S]}{dt}=k_{2}{\left[ES\right]}_{\text{QSS}}$. The QSS value of the complex concentration results from the equation *k*_1_ [*S*] (*E*_tot_ − [*ES*]_QSS_) = (*k*_−1_ + *k*_2_) [*ES*]_QSS_. From this, it follows that *R*([*S*], *E*_tot_) = *k*_2_ *E*_tot_[*S*]/(*k*_*m*_ + [*S*]), where *k*_*m*_ = (*k*_−1_ + *k*_2_)/*k*_1_.

The QE approximation considers that the first reaction of the mechanism is a *fast, reversible reaction*. The simplified mechanism corresponds to a pooling of species. Two pools, *S*_tot_ = [*S*] + [*ES*], and *E*_tot_ = [*E*] + [*ES*] are conserved by the fast reversible reaction, but only one, *E*_tot_ is conserved by the two reactions of the mechanism. The pool *S*_tot_ is slowly consumed by the second reaction and represents the slow variable of the system. The single step approximation reads $S_{tot}\overset{R(S_{tot},E_{tot})}{\to }P,$, or equivalently $\frac{d[P]}{dt}=-\frac{dS_{\text{tot}}}{dt}=k_{2}{\left[ES\right]}_{\text{QE}}$. The QE value of the complex concentration is the unique positive solution of the quadratic equation *k*_1_ (*S*_tot_ − [*ES*]_QE_) (*E*_tot_ − [*ES*]_QE_) = *k*_−1_ [*ES*]_QE_. From this it follows that $R(S_{\text{tot}},E_{\text{tot}})=2k_{2}E_{\text{tot}}S_{\text{tot}}{\left(E_{\text{tot}}+S_{\text{tot}}+k_{-1}/k_{1}\right)}^{-1}{\left(1+\sqrt{1-4E_{\text{tot}}S_{\text{tot}}/{\left(E_{\text{tot}}+S_{\text{tot}}+k_{-1}/k_{1}\right)}^{2}}\right)}^{-1}$. When the concentration of enzyme is small, *E*_tot_ << *S*_tot_, we obtain the original equation of Michaelis and Menten, $R(S_{\text{tot}},E_{\text{tot}})\approx k_{2}\frac{E_{\text{tot}}S_{\text{tot}}}{k_{-1}/k_{1}+S_{\text{tot}}}$.

One of the main difficulties to applying QE or QSS reduction to computational biology models is that QE reactions and QSS species should be specified a priori. For some models, biological information can be used to rank reactions according to their rates. For instance, one knows that metabolic processes and post-transcriptional modifications are more rapid than gene expression. However, this information is rather vague. In detailed gene expression models, some processes can be rapid, while others are much slower. Furthermore, the relative order of these processes can be inverted from one functioning regime to another, for instance the binding and unbinding rates of a repressor to DNA, can be slow or fast depending on various conditions. Even if some numerical approaches such as iterative IM, CSP, and ILDM propose criteria for detecting fast and slow processes, at present there is no general direct method to identify QE reactions and QSS species.

Here we present two methods, based, the first one on singular perturbations, and the second on tropical geometry ideas, allowing to detect QE reactions and QSS species.

The first method uses simulation of the trajectories, therefore, it can only be applied to a fully parametrized model. However, in systems with separation, the sets of QE reactions and QSS species are robust, i.e., remain the same for broad ranges of the parameters. One can use imprecise parameters (resulting for instance from crude estimates or fitting) to compute these sets. The method starts by detecting *slaved species*. Given the trajectories *c*(*t*) of all species, the imposed trajectory of the *i*-th species is a real, positive solution c^*^_i_(*t*) of the polynomial equation

(7) $$P_{i}\left(c_{1}\left(t\right),\ldots ,c_{i-1}\left(t\right),c_{i}^{*}\left(t\right),c_{i+1}\left(t\right),\ldots ,c_{n}\left(t\right)\right)=0,$$

where *P*_*i*_ is the *i*-th component of the rhs of (2). We say that a species *i* is slaved if the distance between the trajectory *c*_*i*_(*t*) and some imposed trajectory c^*^_i_(*t*) is small for some time interval *I*, sup_*t*∈*I*_ | log(*c*_*i*_(*t*)) − log(c^*^_i_(*t*))| < δ, for some δ>0 sufficiently small. The remaining species, that are not slaved, are called slow species.

**Figure 4:**
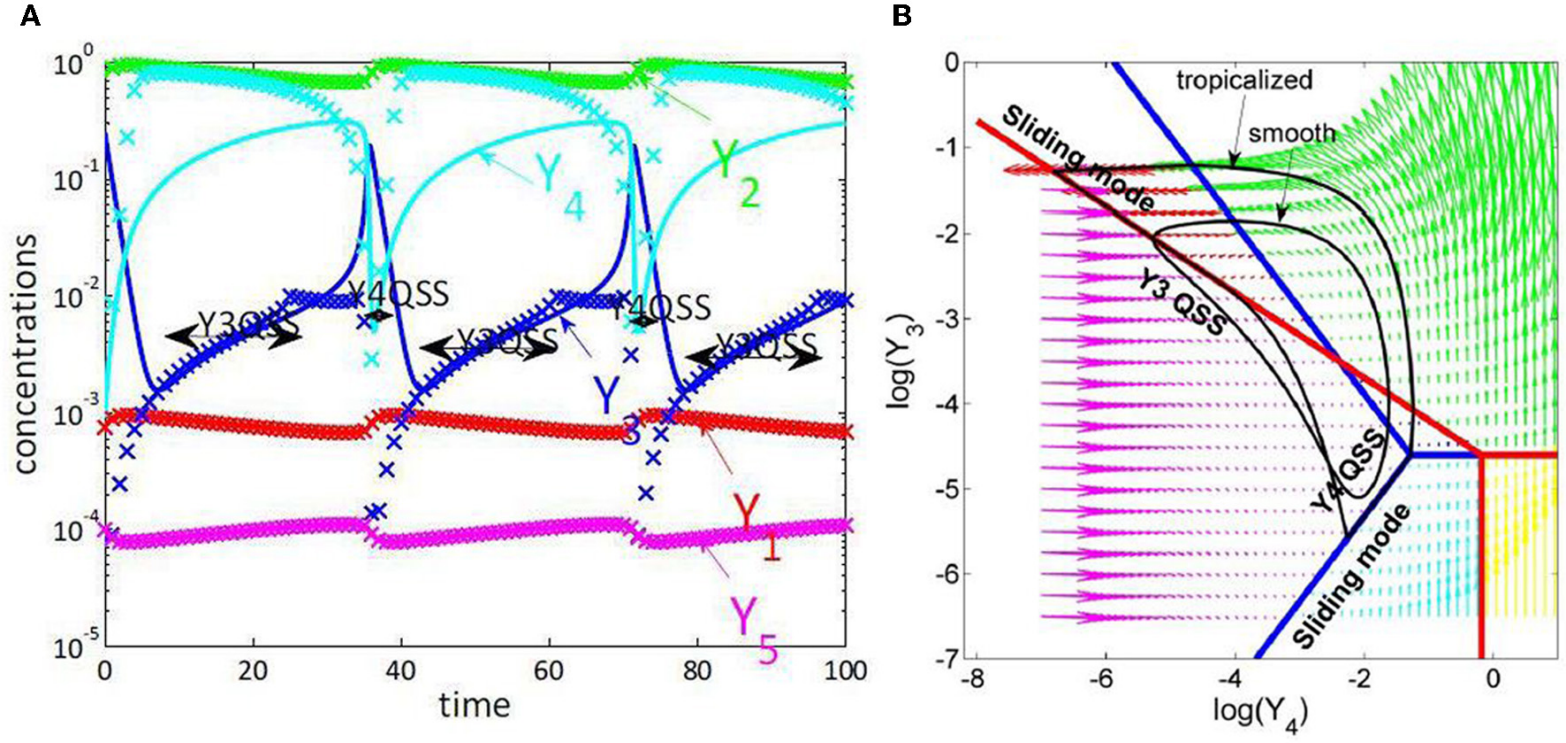
**Tropical geometry and cell cycle modeling.** We considered the five variables cell cycle model defined by the differential equations *y*′_1_ = *k*_9_*y*_2_ − *k*_8_*y*_1_ + *k*_6_*y*_3_, *y*′_2_ = *k*_8_*y*_1_ − *k*_9_*y*_2_ − *k*_3_*y*_2_*y*_5_, *y*′_3_ = *k*′_4_*y*_4_ + *k*_4_*y*_4_*y*^2^_3_/*C*^2^ − *k*_6_*y*_3_, *y*′_4_ = −*k*′_4_*y*_4_ − *k*_4_*y*_4_*y*^2^_3_/*C*^2^ + *k*_3_*y*_2_*y*_5_, *y*′_5_ = *k*_1_ − *k*_3_*y*_2_*y*_5_, proposed in Tyson (1991). **(A)** Comparison of trajectories and imposed trajectories show that variables *y*_1_, *y*_2_, *y*_5_ are always slaved, meaning that the trajectories are close to the 2 dimensional hyperplane defined by the QE condition *k*_8_*y*_1_ = *k*_9_*y*_2_, the QSS condition *k*_1_ = *k*_3_*y*_2_*y*_5_ and the conservation law *y*_1_ + *y*_2_ + *y*_3_ + *y*_4_ = *C*. The variables *y*_3_, *y*_4_ are slaved and the corresponding species are quasi-stationary on intervals. This means that the dimensionality of the dynamics is further reduced to 1, on intervals. **(B)** Tropicalization on logarithmic paper, in the plane of the variables *y*_3_, *y*_4_. The tropical manifold consists of two tripods, represented in blue and red, which divide the logarithmic paper into six polygonal sectors. Monomial vector fields defining the tropicalized dynamics change from one polygonal domain to another. The tropicalized (approximated) and the smooth (not reduced) limit cycle dynamics stay within bounded distance one from another. This distance is relatively small on intervals where the variables *y*_3_ or *y*_4_ are quasi-stationary, which correspond to sliding modes of the tropicalization.

Slaved species are rapid and are constrained by the slow species. The minimum number of variables that we expect for a reduced model is equal to the number of slow species. The slow species can be obtained by direct comparison of the imposed and actual trajectories. This method is illustrated for a model of NFκB canonical pathway in Figure 4.

There are two types of slaved species. Low concentration, slaved species satisfy QSS conditions. Large concentration, slaved species are consumed and produced by fast QE reactions and satisfy QE conditions. Because the reduction schemes are different in the two situations, it is useful to have a method to separate the two cases. Using the values of concentrations can work when concentrations are well separated, but may fail for a continuum of values. A better method is to identify which are the dominant terms in the Equation (7). Using again the example of Michaelis–Menten mechanism, the complex ES will be detected as slaved in both QSS and QE conditions. Equation (7) reads *k*_1_ [*S*] [*E*] = (*k*_−1_ + *k*_2_) [*ES*]. For QE condition, the term *k*_2_ will be dominated by *k*_−1_. We call pruned version of Equation (7) the equation obtained after removing all the dominated monomials, in this case the equation *k*_1_ [*S*] [*E*] − *k*_−1_ [*ES*] = 0. When the pruned version is a combination of reversible reaction rates set to zero, then the slaved species satisfy QE conditions. Again, the comparison of monomials is possible for a fully parametrized model, however we expect this comparison to be robust for models with separation.

The second method to identify QE and QSS conditions, follows from the calculation of the tropicalization (5). This can be done formally and do not require simulation of trajectories and numerical knowledge of the parameters. Indeed, is was shown in Noel et al. that there is a relation between sliding modes of the tropicalized system (5) and the QSS or QE conditions. The system (5) belongs to the class of ordinary differential equations with discontinuous vector fields (Filippov, 1988). In such systems, the dynamics can follow discontinuity hypersurfaces where the vector field is not defined. This type of motion is called sliding mode. When the discontinuity hypersurfaces are smooth and *n* − 1 dimensional (*n* is the dimension of the vector field) then the conditions for sliding modes read:

(8) $$<n_{+}\left(x\right),f_{+}\left(x\right)><0,\;\;\;\;<n\_\left(x\right),f\_\left(x\right)><0,\;\;\;\;x\in \sum ,$$

where *f*_+_, *f*_−_ are the vector fields on the two sides of Σ and *n*_+_ = −*n*_−_ are the interior normals.

In Noel et al. (2012) we have shown the following. If the smooth dynamics obeys QE or QSS conditions and if the pruned polynomial $\tilde{P}$ defining the fast dynamics is a 2-nomial, $\tilde{P_{i}}(c)=a_{1}c^{\alpha_{1}}+a_{2}c^{\alpha_{2}},$ then the QE or QSS equations define a hyperplane of the tropical manifold of $\tilde{P}$, namely *S* = {< log(*c*),α_1_−α_2_> = log (|*a*_1_|/|*a*_2_|)}. The stability of the QE or QSS manifold implies the existence of a sliding mode of the tropicalization (5) along this hyperplane. This result suggests that checking the sliding mode condition (8) on the tropical manifold, provides a method of detecting QE reactions and QSS species.

To illustrate this method, let us use again the Michaelis–Menten example. In this case, two conservation laws allow elimination of two variables *E* and *P* and the dynamics can be described by two ODEs:

(9) $$\begin{array}{c} \frac{d\left[s\right]}{dt}=-k_{1}E_{\text{tot}}\left[S\right]+k_{1}\left[S\right]\left[ES\right]+k_{-1}\left[ES\right]\\ \frac{d\left[ES\right]}{dt}=k_{1}E_{\text{tot}}\left[S\right]-k_{1}\left[S\right]\left[ES\right]-\left(k_{-1}+k_{2}\right)\left[ES\right] \end{array}$$

The tropical manifolds of the two species *S* and *ES* are tripods with parallel arms like in Figure 3. Indeed, the slopes of the arms of tropical manifold are only given by the powers of different variables of the monomials, and these are the same for the two species. Investigation of the flow field close to the tripod arms identifies sliding modes on an unbounded subset *AOB* of the tropical manifold of the species *ES*. This subset is a global attractor of the tropicalized dynamics and represents a tropicalized version of the IM of the smooth system. If the initial data is not in this set, the tropicalized trajectory converges quickly to it and continues on it as a sliding mode. When *k*_2_ >> *k*_−1_, *ES* satisfies QSS conditions leading to the Michaelis–Menten equation. The arm *AO* of the tropical manifold of the species *ES* carry a sliding mode, has the equation *k*_1_*E*_tot_[*S*] = (*k*_−1_ + *k*_2_)[*ES*] >> *k*_1_[*S*][*ES*], and corresponds to the linear regime of the Michaelis–Menten equation. Similarly, the arm *OB* of the tropical manifold of *ES* has the equation *k*_1_*E*_tot_[*S*] = *k*_1_[*S*][*ES*] >> (*k*_−1_ + *k*_2_)[*ES*] and corresponds to the saturated regime of the Michaelis–Menten equation. When *k*_2_ << *k*_−1_, the tropical manifolds of the two species *S* and *ES* practically coincide. Both species are rapid and satisfy QE conditions, namely *k*_1_*E*_tot_[*S*] = *k*_−1_[*ES*] >> *k*_1_ [*S*][*ES*] on the arm *AO*, and *k*_1_*E*_tot_[*S*] = *k*_1_[*S*][*ES*] >> *k*_−1_[*ES*] on the arm *OB*.

The tropicalization can thus be used to obtain global reductions of models. Even when global reductions are not possible (sliding modes leave the tropical manifold or simply do not exist), the tropicalization can be used to hybridize smooth models, i.e., transform them into piecewise simpler models (modes) that change from one time interval to another. These changes occur when the piecewise smooth trajectory of the system meets a hyperplane of the tropical manifold and continues as a sliding mode along this hyperplane or leaves immediately the hyperplane. Hybridization is a particularly interesting approach to modeling cell cycle. Indeed, progression of the cell cycle is a succession of several different regimes (phases). This strategy is illustrated in Figure 4 for a simple cell cycle model.

## 6. Graph rewriting for large non-linear, deterministic, dynamical networks

We have seen in section 3 that model reduction of monomolecular networks with total separation is based on graph rewriting operations.

Similarly, QSS and QE approximations can be used to produce simpler networks from large non-linear networks. The classical implementation of these approximations leads to differential-algebraic equations. It is, however, possible to reformulate the simplified model as a new, simpler, reaction network. We showed in the previous section how to do this for the Michaelis–Menten mechanism under different conditions. In general, one has to solve the algebraic equations corresponding to QE or QSS conditions, eliminate (prune) QSS species and QE reactions, pool reactions (for QSS approximation) or species (for QE approximation), and finally calculate the kinetic laws of the new reactions.

By reaction pooling we understand here replacing a set of reactions by a single reaction whose stoichiometry vector ν is the sum of the stoichiometry vectors ν_*i*_ of the reactions in the pool, ν = ∑_*i*_ γ_*i*_ ν_*i*_. If the reactions are reversible then the coefficients γ_*i*_ can be arbitrary integers, otherwise they must be positive integers. Reaction pools conserve certain species that where previously consumed or produced by individual reactions in the pools. These species were called intermediates in Radulescu et al. (2008). The species that are either produced or consumed by the pools were called terminal in Radulescu et al. (2008). For example, an irreversible chain of reactions *A*_1_ → *A*_2_ → *A*_3_ can be pooled onto a single reaction *A*_1_ → *A*_3_, which in terms of stoichiometry vectors reads $\left[\begin{array}{c} -1\\ 0\\ 1 \end{array}\right]=\left[\begin{array}{c} -1\\ 1\\ 0 \end{array}\right]+\left[\begin{array}{c} 0\\ -1\\ 1 \end{array}\right]$. In this example *A*_1_, *A*_3_ are terminal species and *A*_2_ is an intermediate species. Reaction pooling is used with QSS conditions, in which case the intermediates are the QSS species.

By species pooling we understand replacing a set of species concentrations {*c*_*i*_} by a linear combination with positive coefficients of species concentrations, ∑_*i*_*b*_*i*_*c*_*i*_. Species pooling is used with QE conditions.

In general, the reaction and species pools result from linear algebra. Indeed, let us consider the matrix ***S***^*f*^ that defines the stoichiometry of the rapid subsystem. For the QSS approximation, the matrix ***S***^*f*^ has a number of lines equal to the number of QSS species. The columns of this matrix are the stoichiometries of the reactions in the model, restricted to the QSS species. We exclude zero valued columns, i.e., reactions that do not act on QSS species. For the QE approximation, the number of columns of the matrix ***S***^*f*^ is equal to the number of QE reactions, and the lines of ***S***^*f*^ are the stoichiometries of QE reactions. We exclude zero valued lines corresponding to species that are not affected by QE reactions.

In QE conditions, species pools are defined by vectors in the left kernel of ***S***^*f*^,

(10) $$b^{T}s^{f}=0$$

The vectors *b*, that are conservation laws of the fast subsystem, define linear combinations of species concentrations that are the new slow variables of the system. Of course, one could eliminate from these combinations, the conservation laws of the full reaction network, that will be constant (see Appendix).

In QSS conditions, reaction pools (also called routes) are defined by vectors in the right kernel of ***S***^*f*^,

(11) $$s^{f}\gamma =0$$

According to the definition (11), a reaction pool does not consume or produce QSS species (these are intermediates). One can impose, like in Radulescu et al. (2008), a minimality condition for choosing the reaction pools. A reaction pool is minimal if there is no other reaction pool with less non-zero stoichiometry coefficients. This is equivalent to choosing reaction pools as elementary modes (Von Kamp and Schuster, 2006) of the fast subsystem.

After pooling, QE and QSS algebraic conditions must be solved and the rates of the new reactions calculated. The new rates should be chosen such that the remaining species and pools of species satisfy the simplified ODEs. The choice of the rates is not always unique (some uniqueness conditions are discussed in Radulescu et al. (2008), see also the Appendix). In order to compute the new rates, one has to solve QE and QSS equations. For network with polynomial or rational rates, this implies solving large systems of polynomial equations. The complexity of this task is double exponential on the size of the system (Noel et al., 2011), therefore, one needs approximate solutions. Approximate solutions of polynomial equations can be formally derived when the monomials of these equations are well separated. Some simple recipes were given in Radulescu et al. (2008) and could be improved by the methods of tropical geometry.

**Figure 5:**
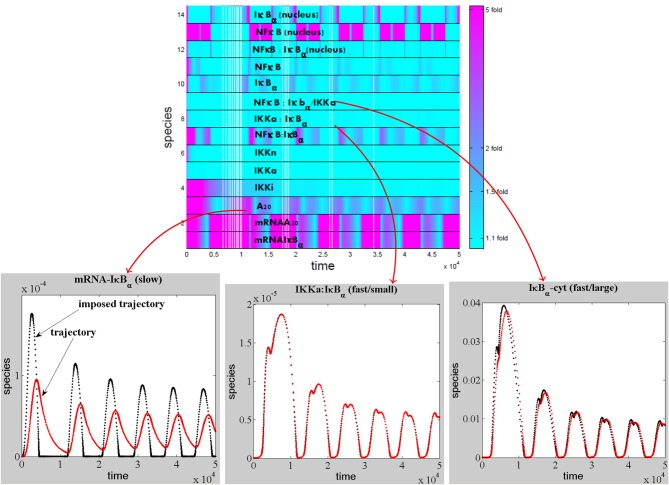
**Detection of the slaved species for a NF-κB pathway model.** The modulus of the log-ratio, |log(*c*_*i*_(*t*)/c^*^_i_(*t*))|, between actual and imposed trajectories has been calculated as a function of time for each species of the model of *NF*κ*B* canonical pathway (proposed in Lipniacki et al., 2004), model ℳ(14, 25, 28) from Radulescu et al. (2008). If the modulus is close to zero (ratio close to one fold from above, or from below) the species is slaved, otherwise the species is slow. Among the slaved species, some have low concentrations and satisfy quasi-steady-state conditions, whereas other have large concentrations and are involved in quasi-equilibrium reactions.

**Figure 6:**
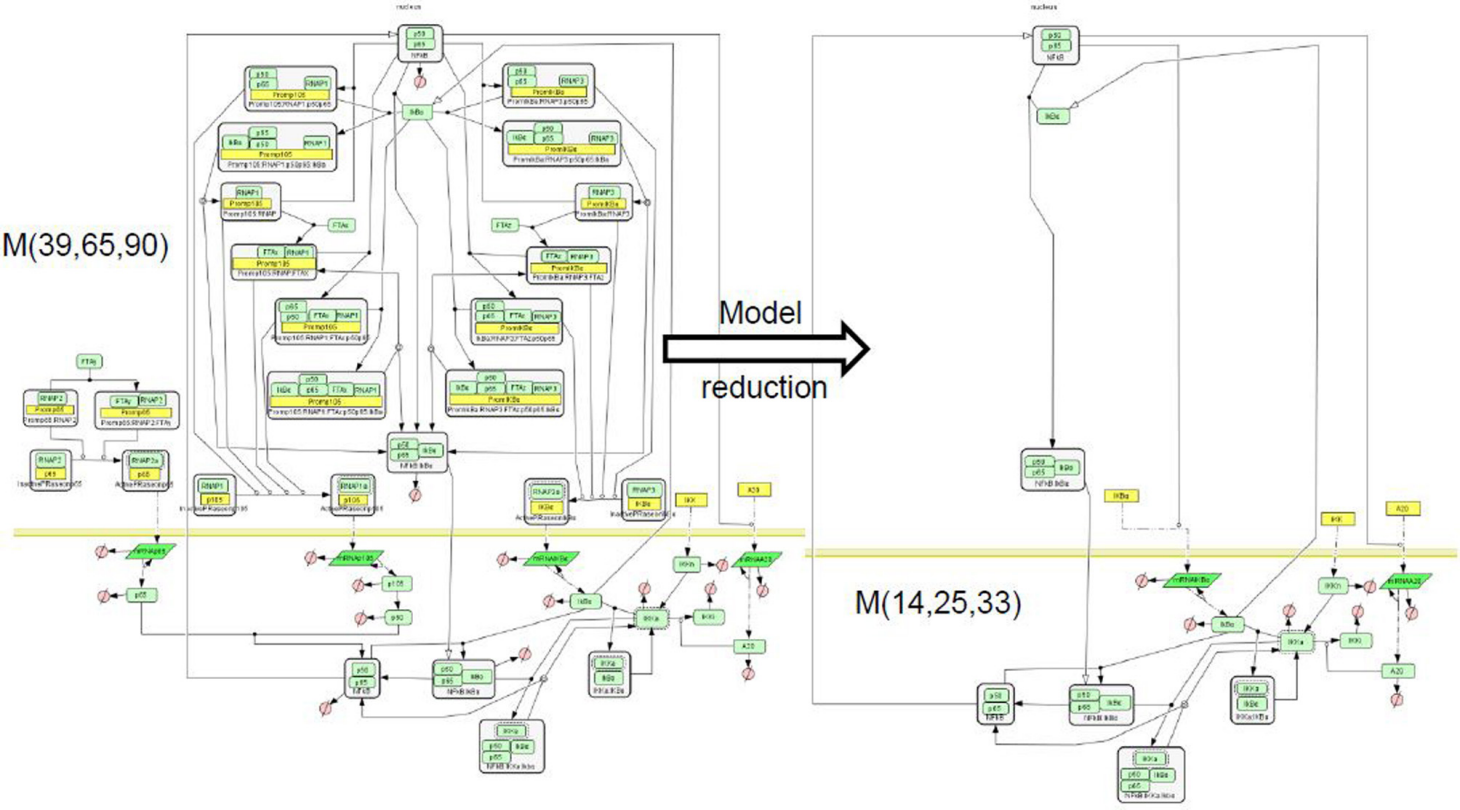
**Reduction of a NF-κB pathway model.** We considered the model of NF-κB signaling [BIOMD0000000227 in Biomodels database (Le Novère et al., 2006), proposing separate production of the subunits p50, p65, the full combinatorics of their interactions as well as with the inhibitor IκB, the positive self-regulation of p50, and in addition an A20 molecule whose production is enhanced upon NF-κB stimulation, and which negatively regulates the activity of the stimulus responding kinase IKK (Radulescu et al., 2008). This model, denoted ℳ(39, 65, 90) contains 39 species, 65 reactions, and 90 parameters. We have reduced it to various levels of complexity. Among the reduced model we obtained one, ℳ(14, 25, 33) that has the same stoichiometry (but different rate functions) as a model published elsewhere by another author (Lipniacki et al., 2004) and denoted ℳ(14, 25, 28) (BIOMD0000000226 in Biomodels database). Incidently, this is also the simplest model in the hierarchy related to ℳ(39, 65, 90). Comparison (not shown) of the rate functions and of the trajectories of the models ℳ(14, 25, 33) and ℳ(14, 25, 28) provided insight into the consequences of various mechanistic modeling choices. The model graphical representation is based on the SBGN standard (Le Novère et al., 2009).

These ideas were used in Radulescu et al. (2008) to reduce several models of NF-κB signaling (Figures 5,6).

The NF-κB activation pathway is complex at many levels. NF-κB is sequestered in the cytoplasm by inactivating proteins named IκB. There are five known members of the NF-κB family in mammals, Rel (c-rel), RelA (p65), RelB, NF-κB1 (p50 and its precursor p105) and NF-κB2 (p52 and its precursor p100). This generates a large combinatorial complexity of dimers, affinities, and transcriptional capabilities. IκB family comprises seven members in mammals (IκBα, IκBβ, IκBε, IκBγ, Bcl-3). All these inhibitors display different affinities for NF-κB dimers, multiplying the combinatorial complexity. The activation of NF-κB upon signaling, occurs by phosphorylation by a kinase complex, then ubiquitination, and finally degradation of IκB molecules. The activation signal is transmitted by several possible pathways most of them activating the kinase IKK that modifies IκB. In the canonical pathway, one important determinant of IKK dynamics is the protein A20 that inhibits IKK activation. A20 expression is controlled by NF-κB. In order to cope with this complexity a model containing 39 species, 65 reactions, and 90 parameters was proposed in Radulescu et al. (2008). Of course, not all reactions and parameters of this complex model are important. In order to determine, in a rational and systematic way, which of the model features are critical, we have used model reduction.

Graph rewriting was performed in a modular way, by applying the pruning and pooling rules to tightly connected submodels of the NF-κB network. The computation of the reaction pools was performed using Matlab and METATOOL (Von Kamp and Schuster, 2006). Using submodel decomposition reduces the complexity of computing elementary modes and of solving large systems of algebraic equations needed for recalculating the reaction rates.

To give an example of modular reduction, let us consider the set of reactions involving six cytoplasmic located intermediates (IKK|active, IKK|inactive, IKK, IKK|active:IkBa, IKK|active:IkBa:p50:p65, p50:p65@csl) and four terminal species (A20, IkBa@csl, IkBa:p50:p65@csl, p50:p65@ncl). As can be seen from Figure 5, the six intermediate species are slaved. The reactions of this submodel form the cytoplasmic part of the signaling mechanism, including 11 kinase transformation reactions, a complex release reaction, a complex formation reaction, and the NF-κB translocation reaction. The elementary modes of the submodel [computed using METATOOL (Von Kamp and Schuster, 2006)] are used to define the reactions pools. For this submodel, we find two elementary modes, that can be described as the modulated inhibitor degradation (IkBa@csl → ∅), and a reaction summarizing the NF-κB release and translocation (IkBa:p50:p65@csl → p50:p65@ncl), respectively. In order to compute the reaction rates of the two elementary modes as functions of the concentrations of the terminal species, we find approximate solutions of the QSS equations for the intermediate species and equate, for the variation rates of each terminal species, the contributions of elementary modes to the total known variation rate in the unreduced model (see Appendix). The two rates are *k*_21*p*1_ [*IkBa*@*csl*] [*IkBa*:*p*50:*p*65@*csl*]/((*k*_21*p*2_ + [*IkBa*@*csl*])(*k*_21*p*3_+ [*A*20])) for the modulated inhibitor degradation, and *k*_15*p*1_ [*IkBa*:*p*50:*p*65@*csl*]/((*k*_15*p*2_ + [*IkBa*@*csl*])(*k*_15*p*3_+ [*A*20])) for the release and translocation reaction.

## 7. Model reduction and model identification

Computational biology models contain mechanistic details that can not all be identified from available experimental data. Determining the parameters of such complex models could lead to overfitting, describing noise, rather than features of data, or can be simply meaningless, when model behavior is not sensitive to the parameters. Furthermore, many model identification methods (Golightly and Wilkinson, 2011) suffer from the “curse of dimensionality” as it becomes increasingly difficult to cover the parameter space when the number of parameters increases. A rather efficient strategy to bypass these problems is to use model reduction. This method is known in machine learning as backward pruning or post-pruning (Witten and Frank, 2005). It consists in finding a complex model that fits data well and then prune it back to a simpler one that also fits the data well. Far from being redundant, backward pruning can be successfully used in computational biology. Rather often, one starts with a complex model coping with mechanistic details of the network regulation. Then, over-fitting and problems of identifiability of the parameters are avoided by model reduction. By model reduction, the mechanistic model is mapped onto a simpler, phenomenological model. For instance, gene transcription and translation can be represented as one step and one constant in a phenomenological model, but can consist of several steps such as initiation, transcription of mRNA leading region, ribosome binding, translation, folding, maturation, etc., in a complex model. Not all of these steps are important for the network functioning and not all parameters are identifiable from the observed quantities. Following reduction, the inessential steps are pruned and several sensitive parameters are compacted into a few effective parameters that are identifiable.

As discussed in Radulescu et al. (2006, 2008); Radulescu and Gorban (2009); Ferguson et al. (2012), model reduction unravels the important features and the sensitive parameters of the model.

Using model reduction for determining critical features of the model has many advantages relative to numerical sensitivity studies (Rabitz et al., 1983; Ihekwaba, 2004; Gunawan et al., 2005). This approach is less time consuming, brings more insight, and is based on qualitative comparison of the order of the parameters and therefore does not need exhaustive scans of parameter values. In the applications described in Radulescu et al. (2006, 2008); Radulescu and Gorban (2009); Ferguson et al. (2012), the sensitive parameters of the pruned model are combinations (most often monomials) of the parameters of the complex models. As only the sensitive combinations can be fitted from data, it is important to have estimates of some individual parameters, allowing to determine the remaining ones.

**Figure 7:**
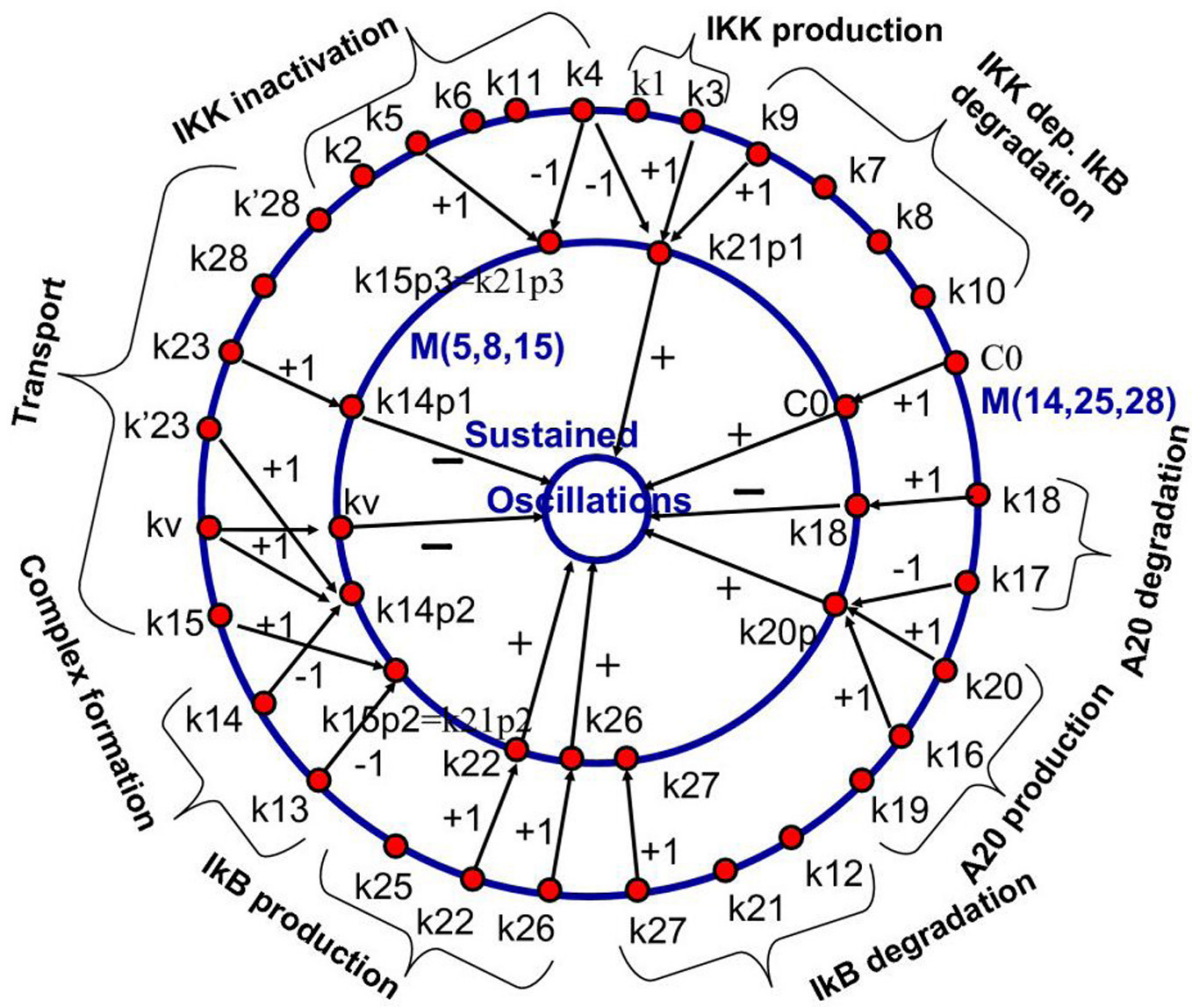
**Backward pruning strategy.** The model ℳ(14, 25, 28) from Radulescu et al. (2008) (first proposed in Lipniacki et al. (2004), see also BIOMD0000000226 in Biomodels database) was used to generate a hierarchy of simpler models, the simplest one being ℳ(5, 8, 15). We show the mapping between the parameters of the models ℳ(14, 25, 28) and ℳ(5, 8, 15). Parameters of the first model are gathered into monomials that are parameters of the reduced model. The integers on the arrows connecting parameters represent the corresponding powers of the parameters in the monomial. The innermost circle represents a dynamical property of the model that is influenced positively, negatively, or negligibly by the effective parameters (parameters of the reduced model). From Radulescu et al. (2008).

This methodology has been first proposed in Radulescu et al. (2008). The model reduction of the NF-κB model in Radulescu et al. (2008) leads to new, effective parameters that are monomials of the parameters of the complex model. The correspondence between the initial parameters and the effective parameters is shown in Figure 7. Although not fully exploited in the theoretical study (Radulescu et al., 2008), this mapping can be used for model parameter identification. Effective parameters have increased observability and could be obtained from experimental data. The values of the effective parameters can be used to constrain the parameters of the full model. Some of the parameters of the full model, that are not sensitive or contribute to effective parameters together with other parameters remain arbitrary and could be fixed to generic values.

## 8. Conclusion

The mathematical techniques described in this paper define strategies for the study of large dynamical network models in computational biology. Large networks are needed in order to understand context dependence, specialization, and individuality of the cell behavior. Extensive pathway database accumulation supports somehow the idea that biological cell is a puzzle of networks and pathways, and that once these are put together in a tightly bound, coherent map, the cell physiology should be unraveled by a computer simulation. Actually, confronting biochemical networks with real life is not an easy challenge. Model reduction techniques are needed to bring us one step closer to this objective, as these methods can reveal critical features of complex organizations.

We have proposed that the ideas of limitation and dominance are fundamental for understanding computational biology dynamical models. The essential, critical features of systems with many separated time scales, can be resumed by a dominant, reduced, subsystem. This dominant subsystem depends on the order relations between model parameters or combinations of model parameters. We have shown how to calculate such a dominant subsystem for linear and non-linear networks. Geometrical interpretation of these concepts in terms of tropicalization provides a powerful framework, allowing to identify IM, QSS species, and QE reactions. We have also discussed how model reduction can be applied to backward pruning parameter learning strategies.

Future efforts are needed to extend these mathematical ideas and model reduction algorithms and implement them into computational biology tools.

### Conflict of interest statement

The authors declare that the research was conducted in the absence of any commercial or financial relationships that could be construed as a potential conflict of interest.

## Appendix: algorithms

### Algorithm 1: reduction of monomolecular networks with separation

This algorithm consists of three procedures.

#### I. Constructing of an auxiliary reaction network: pruning

For each *A*_*i*_ branching node (substrate of several reactions) let us define κ_*i*_ as the maximal kinetic constant for reactions *A*_*i*_ → *A*_*j*_: κ_*i*_ = max_*j*_ {*k*_*ji*_}. For correspondent *j* we use the notation ϕ(*i*): ϕ(*i*) = arg max_*j*_ {*k*_*ji*_}.

An auxiliary reaction network V is the set of reactions obtained by keeping only *A*_*i*_ → *A*_ϕ(*i*)_ with kinetic constants κ_*i*_ and discarding the other, slower reactions. Auxiliary networks have no branching, but they can have cycles and confluences. The correspondent kinetic equation is

(12) $${\dot{c}}_{i}=-\kappa_{i}c_{i}+\sum_{\phi (j)=i}\kappa_{i}c_{i},$$

If the auxiliary network contains no cycles, the algorithm stops here.

#### II. Gluing cycles and restoring cycle exit reactions

In general, the auxiliary network V has several cycles *C*_1_, *C*_2_, … with lengths τ_1_, τ_2_, …>1.

These cycles will be “glued” into points and all nodes in the cycle *C*_*i*_, will be replaced by a single vertex *A*^*i*^. Also, some of the reactions that were pruned in the first part of the algorithm are restored with renormalized rate constants. Indeed, reaction exiting a cycle are needed to render the correct dynamics: without them, the total mass accumulates in the cycle, with them the mass can also slowly leave the cycle. Reactions *A* → *B* exiting from cycles (*A* ∈ *C*_*i*_, *B* ∉ *C*_*i*_) are changed into *A*^*i*^ → *B* with the rate constant renormalization: let the cycle *C*^*i*^ be the following sequence of reactions *A*_1_ → *A*_2_ → … *A*_τ_*i*__ → *A*_1_, and the reaction rate constant for *A*_*j*_ → *A*_j+1_ is *k*_*j*_ (*k*_τ_*i*__ for *A*_τ_*i*__ → A_1_). The quasi-stationary normalized distribution in the cycle is:

$$c_{j}^{ο}=\frac{1}{k_{j}}{\left(\sum_{j=1}^{\tau_{i}}\frac{1}{k_{j}}\right)}^{-1},j=1,\ldots ,\tau_{i}.$$

The reaction *A*_*j*_ → *B* (*A* ∈ *C*_*i*_, *B* ∉ *C*_*i*_) with the rate constant *k* is changed into *A*^*i*^ → *B* with the rate constant *c*^o^_*j*_ *k*.

Let the cycle *C*_*i*_ have the limiting steps that is much slower than other reactions. For the limiting reaction of the cycle *C*_*i*_ we use notation *k*_lim *i*_. In this case, *c*^o^_*j*_ = *k*_lim *i*_/*k*_*j*_. If *A* = A_*j*_ and *k* is the rate constant for *A* → *B*, then the new reaction *A*^*i*^ → *B* has the rate constant *kk*_lim *i*_/*k*_*j*_. This rate is obtained using quasi-stationary distribution for the cycle.

The new auxiliary network $V^{1}$ is computed for the network with glued cycles. Then we prune it, extract cycles, glue them, iterate until a acyclic network is obtained $V^{m}$, where *m* is the number of iterations.

#### III. Restoring cycles

The previous procedure gives us the sequence of networks $V^{1},\ldots ,V^{m}$ with the set of vertices $A^{1},\ldots A^{m}$ and reaction rate constants defined for each $V^{i}$ in the processes of pruning and gluing.

The dynamics of species inside glued cycles is lost after their gluing. A full multi-scale approximation (including relaxation inside cycles) can be obtained by restoration of cycles. This is done starting from the acyclic auxiliary network $V^{m}$ back to $V^{1}$ through the hierarchy of cycles. Each cycle is restored according to the following procedure:

- We start the reverse process from the glued network $V^{m}$ on $A^{m}$. On a step back, from the set $A^{m}$ to $A^{m-1}$ and so on, some of glued cycles should be restored and cut. On the *q*th step we build an acyclic reaction network on the set of vertices $A^{m-q}$, the final network is defined on the initial vertex set and approximates relaxation of the initial networks.
- To make one step back from $V^{m}$ let us select the vertices of $A^{m}$ that are glued cycles from $V^{m-1}$. Let these vertices be *A*^*m*^_1_, *A*^*m*^_2_, …. Each *A*^*m*^_*i*_ corresponds to a glued cycle from $V^{m-1}$, *A*^*m*−1^_*i*1_ → *A*^*m*−1^_*i*2_ → … *A*^*m*−1^_*i* τ_*i*__ → *A*^*m*−1^_*i*1_, of the length τ_*i*_. We assume that the limiting steps in these cycles are *A*^*m*−1^_*i*τ_*i*__ → *A*^*m*−1^_*i*1_. Let us substitute each vertex *A*^*m*^_*i*_ in $V^{m}$ by τ_*i*_ vertices *A*^*m*−1^_*i*1_, *A*^*m*−1^_*i*2_, … *A*^*m*−1^_*i*τ_*i*__, and add to $V^{m}$ reactions *A*^*m*−1^_*i*1_ → *A*^*m*−1^_*i*2_ → … *A*^*m*−1^_*i* τ_*i*__ (that are the cycle reactions without the limiting step) with corresponding constants from $V^{m-1}$.
- If there exists an outgoing reaction *A*^*m*^_*i*_ → *B* in $V^{m}$ then we substitute it by the reaction *A*^*m*−1^_*i*τ_*i*__ → *B* with the same constant, i.e., outgoing reactions *A*^*m*^_*i*_ → … are reattached to the heads of the limiting steps. Let us rearrange reactions from $V^{m}$ of the form *B* → *A*^*m*^_*i*_. These reactions have prototypes in $V^{m-1}$ (before the last gluing). We simply restore these reactions. If there exists a reaction *A*^*m*^_*i*_ → *A*^*m*^_*j*_ then we find the prototype in $V^{m-1}$, *A* → *B*, and substitute the reaction by *A*^*m*−1^_*i*τ_*i*__ → *B* with the same constant, as for *A*^*m*^_*i*_ → *A*^*m*^_*j*_.
- After the previous step is performed, the vertices set is $A^{m-1}$, but the reaction set differs from the reactions of the network $V^{m-1}$: the limiting steps of cycles are excluded and the outgoing reactions of glued cycles are included (reattached to the heads of the limiting steps). To make the next step, we select vertices of $A^{m-1}$ that are glued cycles from $V^{m-2}$, substitute these vertices by vertices of cycles, delete the limiting steps, attach outgoing reactions to the heads of the limiting steps, and for incoming reactions restore their prototypes from $V^{m-2}$, and so on.

After all, we restore all the glued cycles, and construct an acyclic reaction network on the set A. This acyclic network approximates relaxation of the initial network. We call this system the *dominant system*.

Note that the reduction algorithm does not need precise values of the constants. It is enough to have an initial ordering of the constants. Then, the auxiliary network is obtained only from this ordering. However, after a first iteration, and if the initial network contains cycles, some of the exit constant are renormalized and the new rate constants become monomials of the old ones. In order to prune again, we need to compare these monomials. Monomials of well separated constants are generically well separated (Gorban and Radulescu, 2008). However, a freedom remains on ordering these new monomials, leading to several possible reduced acyclic digraphs, given an initial digraph with ordering of the constants (Figure 1 of the main text).

### Algorithm 2: reduction of non-linear networks with separation

This algorithm consists of the following procedures.

#### I. Identification of QSS species and QE reactions

There are two methods of identification, trajectory-based, and tropicalization-based. Presently we are using the trajectory based method.

***Detect slaved species.*** After generating trajectories *c*(*t*) for *t* ∈ *I*, for each species compute the distances δ_*i*_ = sup_*t* ∈ *I*_ |log(*c*_*i*_(*t*))−log(*c*^*^_*i*_(*t*))|. Use k-means clustering to separate species into two groups, slaved (small values of δ) and slow (large values of δ) species.

***Prune.*** For each *P*_*i*_ (polynomial rate) corresponding to slaved species, compute the pruned version $\tilde{P_{i}}$ by eliminating all monomials that are dominated by other monomials of *P*_*i*_.

***Identify QE reactions and QSS species.*** Identify, in the structure of $\tilde{P_{i}}$ the forward and reverse rates of QE reactions. This step can be performed by recipes presented in Soliman and Heiner (2010). The slaved species not involved in QE reactions are QSS.

#### II. Exploiting QSS conditions, pruning intermediate species, pooling reactions

***Define subsets and matrices.*** Given the set of QSS (intermediate) species *I*, one defines the set ${ℛ}_{I}$ of reactions acting on them. The terminal species *T*, are the other species, different from *i*, on which act the reactions from ${ℛ}_{I}$. Define two stoichiometric matrices ***S***^*f*^ and ***S***^*T*^. ***S***^*f*^ defines the fast subsystem and has a number of lines equal to the number of QSS species, and a number of columns equal to the number of reactions ${ℛ}_{I}$. ***S***^*T*^ contains the stoichiometries of the terminal species for the same reactions ${ℛ}_{I}$. Species *I* will be pruned, and reactions ${ℛ}_{I}$ will be pooled.

***Compute elementary modes (EMs).*** Compute elementary modes of non-zero terminal stoichiometry as minimal solutions of ***S***^*f*^ γ = 0, ***S***^*T*^ γ ≠ 0, the minimality being defined with respect to the number of non-zero coefficients. ***S***^*T*^ γ ≠ 0 on the output of elementary modes packages such as METATOOL. If the terminal stoichiometries of the EMs are dependent, restrict to a subset of independent terminal stoichiometries.

***Solve QSS equations.*** Find approximate formal solutions for systems of QSS algebraic equations. This step is not yet automatic. It will be automatized in subsequent work by using tropical geometry methods.

***Find rates of EMs.*** To each elementary mode γ_*i*_, associate a kinetic law giving the rate of the EM as a function of the terminal species concentrations *R*^*^_*i*_(*c*_*T*_). Let *R*(*c*_*T*_) be the vector of rates of reactions in ${ℛ}_{I}$. The dependence of these rates on *c*_*T*_ is direct, or indirect, via *c*_*I*_ that can be now expressed as function of *c*_*T*_. Then, the EM rates *R*^*^_*i*_(*c*_*T*_) must satisfy ***S***^*T*^ ***R***(*c*_*T*_) = ∑ ***R***^*^_*i*_(*c*_*T*_)***S***^*T*^γ_*i*_. This equation has an unique solution if the vectors ***S***^*T*^γ_*i*_ are independent (this justifies the independence condition for the terminal stoichiometries of EMs).

#### III. Exploiting QE conditions, pruning QE reactions, pooling species

***Define subsets and matrices.*** Given the set of QE reactions *Q*, one defines the set *S* of species that are affected by them. The species *S* are also affected by other reactions that we call terminal, *Q*_*T*_. Define two stoichiometric matrices ***S***^*f*^ and ***S***^*T*^. ***S***^*f*^ defines the fast subsystem and has a number of lines equal to the cardinal of *E*, and a number of columns equal to the cardinal of *Q*. ***S***^*T*^ contains the stoichiometries of the reactions *Q*_*T*_ for the same species *S* (it has the same number of lines as ***S***^*f*^). Reactions *Q* will be pruned and species *E* will be pooled.

***Compute species pools.*** Species pools are computed as minimal solutions of ***bS***^*f*^ = 0, ***bS***^*T*^ ≠ 0 (the second condition stands for looking for conservation laws of the fast subsystem that are not conserved by the entire network; the minimality condition means that we compute elementary modes of the transpose matrix ***S***^*f*^).

***Solve QE equations.*** Same methods as for QSS conditions. Solve the QSS equations together with the conservation of pools and express the concentrations of the species *E* as functions of the pools *c*^*^_*i*_ = <***b***_*i*_, ***c*** >.

***Find new rates.*** Re-express (by substitution) the rate of each reaction from *Q*_*T*_ in terms of pools *c*^*^_*i*_.
